# Modeling present and future distribution of plankton populations in a coastal upwelling zone: the copepod *Calanus chilensis* as a study case

**DOI:** 10.1038/s41598-023-29541-9

**Published:** 2023-02-23

**Authors:** Reinaldo Rivera, Rubén Escribano, Carolina E. González, Manuela Pérez-Aragón

**Affiliations:** 1 Millennium Institute of Oceanography (IMO), University of Concepcion, 4030000 Concepcion, Chile; 2 Department of Oceanography, Faculty of Natural and Oceanographic Sciences, University of Concepcion, 4030000 Concepcion, Chile

**Keywords:** Biogeography, Ecological modelling, Macroecology, Marine biology, Climate change

## Abstract

Predicting species distribution in the ocean has become a crucial task to assess marine ecosystem responses to ongoing climate change. In the Humboldt Current System (HCS), the endemic copepod *Calanus chilensis* is one of the key species bioindicator of productivity and water masses. Here we modeled the geographic distribution of *Calanus chilensis* for two bathymetric ranges, 0–200 and 200–400 m. For the 0–200 m layer, we used the Bayesian Additive Regression Trees (BART) method, whereas, for the 200–400 m layer, we used the Ensembles of Small Models (ESMs) method and then projected the models into two future scenarios to assess changes in geographic distribution patterns. The models were evaluated using the multi-metric approach. We identified that chlorophyll-a (0.34), Mixed Layer Depth (0.302) and salinity (0.36) explained the distribution of *C. chilensis*. The geographic prediction of the BART model revealed a continuous distribution from Ecuador to the southernmost area of South America for the 0–200 m depth range, whereas the ESM model indicated a discontinuous distribution with greater suitability for the coast of Chile for the 200–400 m depth range. A reduction of the distribution range of *C. chilensis* is projected in the future. Our study suggests that the distribution of *C. chilensis* is conditioned by productivity and mesoscale processes, with both processes closely related to upwelling intensity. These models serve as a tool for proposing indicators of changes in the ocean. We further propose that the species *C. chilensis* is a high productivity and low salinity indicator at the HCS. We recommend further examining multiple spatial and temporal scales for stronger inference.

## Introduction

The knowledge and understanding of oceanographic drivers influencing patterns of distribution of zooplankton over large-scale domains, such as ocean basins, are poor, limiting our capacity to predict changes in diversity and spatial distribution under a changing ocean^1,2^. The species distribution of species forming the zooplankton is known to be affected by changes in oceanographic conditions, such as oxygenation, temperature, salinity, and stratification^3,4^. This reveals the strong dependence of these organisms on oceanographic variables due to their limited migration capacity, which reflects the strong effects of hydrographic patterns on their distribution^3,5^. Specifically, marine copepods respond to changes in oceanic environmental conditions, and thus might act as indicators of natural perturbations affecting the whole system^6^.

The Humboldt Current System (HCS) is recognized as one of the most productive areas of the world, sustaining a high production of pelagic fishes^6^, closely associated with upwelling events driven by wind at different intensities and frequencies along the South American coast^7^. The HCS is characterized by a predominant northward flow of surface waters of subantarctic origin and by a strong upwelling of cold, nutrient-rich subsurface waters of equatorial origin^8^. These dynamic and heterogeneous conditions of HCS support a high diversity of species^8,9^.

Within this large ecosystem, one of the most characteristic species is the planktonic copepod *Calanus chilensis* (Brodsky, 1959) (Calanoida:Calanidae), an endemic species^6^ and a typical herbivorous copepod^6^. It is distributed from ~ 1 to 23°S and is one of the most abundant species among copepods^10–13^. However, in the last decades, observations show *C. chilensis* is being replaced by *C. australis* toward oceanic waters^14^. Additionally, *C. chilensis* is associated with upwelling centers, reproducing continuously throughout the year^12,15–17^, and showing high abundance near the coast at the upper 250 m of the water column, which coincides with the Poleward Undercurrent^13^.

Given its high abundance and extensive geographic distribution, *C. chilensis* appears to be an important contributor to secondary production and thus a crucial link between primary production and fish production^6,18^. For its prominent ecological role, *C. chilensis* is recognized as key species worldwide in terms of secondary production, and this has motivated considerable research on its life cycle and secondary productivity^6,12,13^. Despite its wide latitudinal distribution, information on its geographic distribution is fragmentary^19^, and even scarce in relation to the environmental drivers that explain its wide geographic distribution. This is certainly basic knowledge for evaluating the importance of this species to the production of the whole system^6^. Additionally, global climate change is driving changes in the phenology, distribution and abundance of species^20–22^, with a significant impact on marine ecosystems^23^. Nevertheless, for many taxa, there is a limited understanding of how geographic distribution patterns will be affected or changed^21^. Therefore, predicting potential habitat changes would require knowing how global climate change may affect populations and communities in the medium and long term.

Given this background, *C. chilensis* appears to provide an opportunity to test hypotheses on the response of pelagic populations to oceanographic conditions^6^. One way to assess its patterns, causes and consequences is the use of the wide availability of reliable biological and environmental information, which enables the generation of predictive models of the geographic distribution, as well as allows to evaluate hypotheses and generate predictive models of the geographic distribution of the species^24,25^. The current availability of global environmental databases (e.g. Copernicus, bio-Oracle), geo-referenced records of species (e.g. OBIS, GBIF), and the development of the geographic information system (GIS) have allowed significant advances in the study and testing of hypotheses related to the spatial distribution of species using new approaches and ecological models^25,26^. Among these tools, species distribution modeling (SDM) that uses spatially explicit information has been widely implemented to study the distribution patterns and environmental factors that explain and predict the distribution of species and populations^27,28^, in the present as well as in other temporal scenarios^29^.

For the marine environment, there are numerous examples of SDM implemented to answer questions regarding current species distribution patterns^30–32^, bycatch^33,34^, marine conservation planning^35,36^, range shifts^37,38^, biological invasions^39–41^ and climate change^42,43^ (see Ref.^44^ for recent reviews). Nevertheless, fish, mollusks and mammals are the main taxa studied^44^. The latter denotes that pelagic organisms such as zooplankton are less studied in relation to vertebrates and invertebrates at a macroscale^1,45–49^.

The lack of macro-scale studies on zooplankton makes evident the scarce knowledge of the patterns and factors modulating the distribution of organisms that due to their trophic position and/or their status as indicators of oceanic conditions are considered ecologically relevant^13,50^. Particularly, a macroscale change in temperature has modified the geographical range of some copepod species^3,51,52^. For this reason, knowing the mechanisms that regulate biogeographical patterns at the macroscale allow to predict variation in species distribution under the effect of natural or anthropogenic impacts^53,54^. Therefore, under this background, the HCS may constitute a suitable model to improve our understanding of underlying mechanisms modulating distribution patterns of species with high indicator values such as *C. chilensis*.

**Figure 1:**
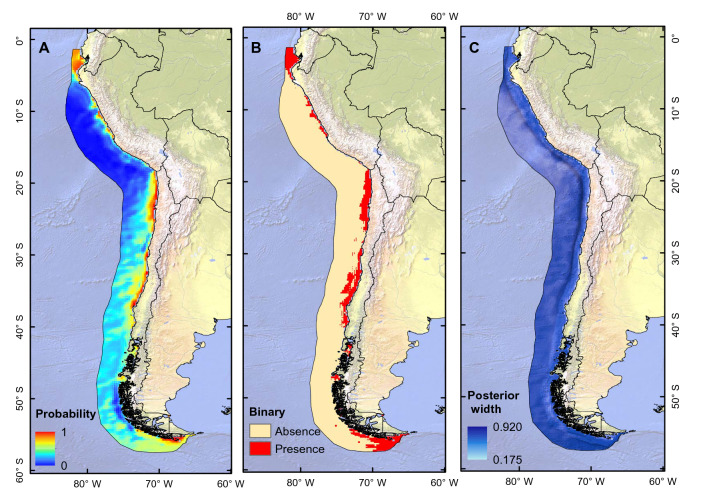
Potential current distribution maps generated by BART approach for the 0–200 m depth range. (**A**) Median of the posterior probability of the presence of *C. chilensis*, (**B**) binary model, and (**C**) posterior width (95% credible interval). This figure was generated using ArcGIS 10.4.1 (ESRI, Redland, CA; www.esri.com). Map projection is WGS84 (EPSG 4326).

In this research, we assessed the distribution of the copepod species *C. chilensis* in the HCS and the environmental drivers shaping it, under present climatic conditions and under projected global climate change. We thus aimed at understanding the influence of oceanographic conditions over large-scale patterns and evaluating their value for an indicator species in the HCS. For the estimation of environmental effects, we used *C. chilensis* occurrence data and environmental variables to predict the potential geographic distribution of this species and identify its drivers, by applying Bayesian Additive Regression Trees (BART) and ensembles of small models (ESM) for two distinct bathymetric ranges (see Fig. 1 and Fig. 3). The models were projected under a climate change scenario to study the geographic distribution of this species in the future.

## Results

### 0–200 m depth range

**Figure 2:**
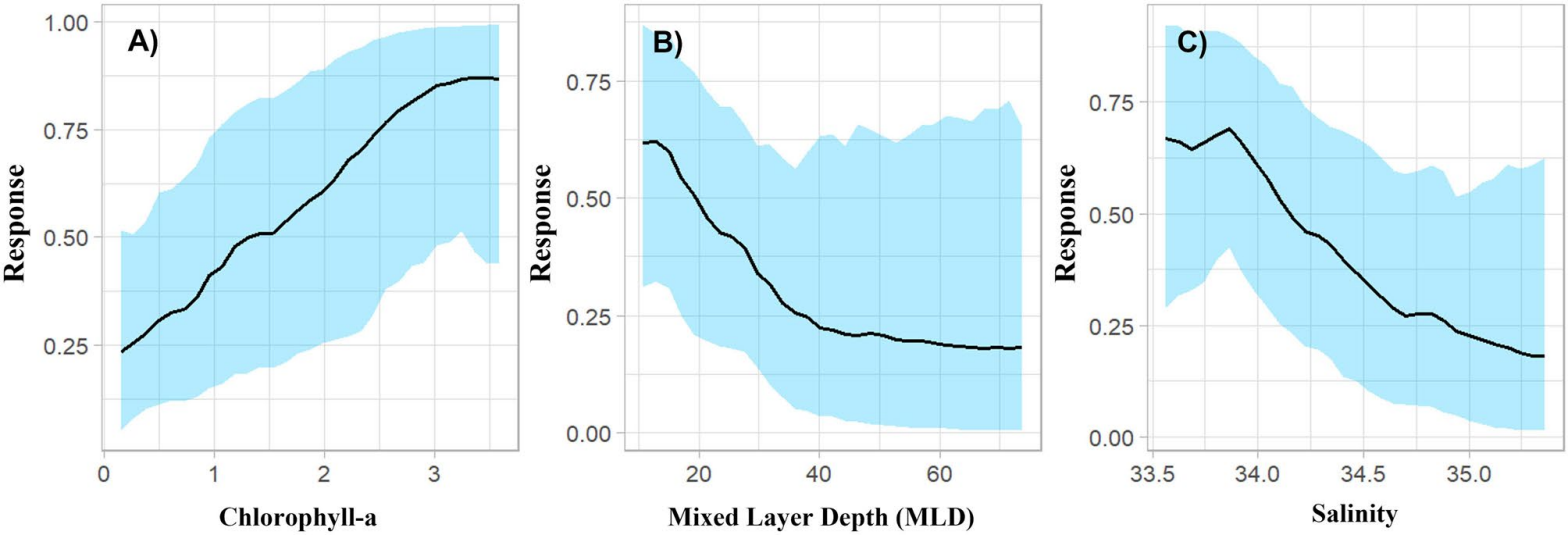
Partial dependence plot which shows the effect of each variable on the probability of presence of *C. chilensis* (**A**) chlorophyll-a, (**B**) mixed layer depth, and (**C**) salinity. Light blue = 95% credible interval. 0–200 m depth range. Figure done using R (https://www.r-project.org).

The geographic prediction of the BART model in the 0–200 m depth range using the Copernicus database presented a high performance [Area Under the Curve (AUC) = 0.933; True Skill Statistic (TSS) = 0.783]. The threshold that maximizes TSS was 0.446, and it was used as the threshold to generate the binary prediction. The variables that contributed most to the predicted distribution of *C. chilensis* were chlorophyll-a (0.34), Mixed Layer Depth [MLD (0.302)], and salinity (0.36). The geographical prediction of the model indicates high probability of presence mostly near the coasts from the northern region of the HCS (off Ecuador) to Tierra del Fuego (off southern Chile/Argentina), with an increase in the predicted probability in oceanic areas between 30 and 41° S (Fig. 1A). The binary prediction revealed a continuous longitudinal strip of predicted presence from Ecuador (1° S) to the southernmost part of Chile and Argentina (56° S) (Fig. 1B). The credible interval width (measure of spatial uncertainty) indicates that the highest uncertainty of prediction is located at the geographic extremes of the HCS, being more pronounced in front of the Magallanes region (Fig. 1C). The partial dependence curves revealed that chlorophyll-a has a positive effect on the presence of *C. chilensis* (Fig. 2A). On the other hand, the probability of presence decreases at higher values of MLD (Fig. 2B) and salinity, with lower probability of presence of *C. chilensis* from values over 34 psu (Fig. 2C).

The BART model approach for the 0–200 m depth using the bio-ORACLE database revealed an AUC of 0.972 and TSS of 0.875. The geographical prediction indicated that the highest probability of presence is located continuously from ~ 17° to 41° S, mainly in coastal areas of the HCS (Fig. S1).

### 200–400 m depth range

**Figure 3:**
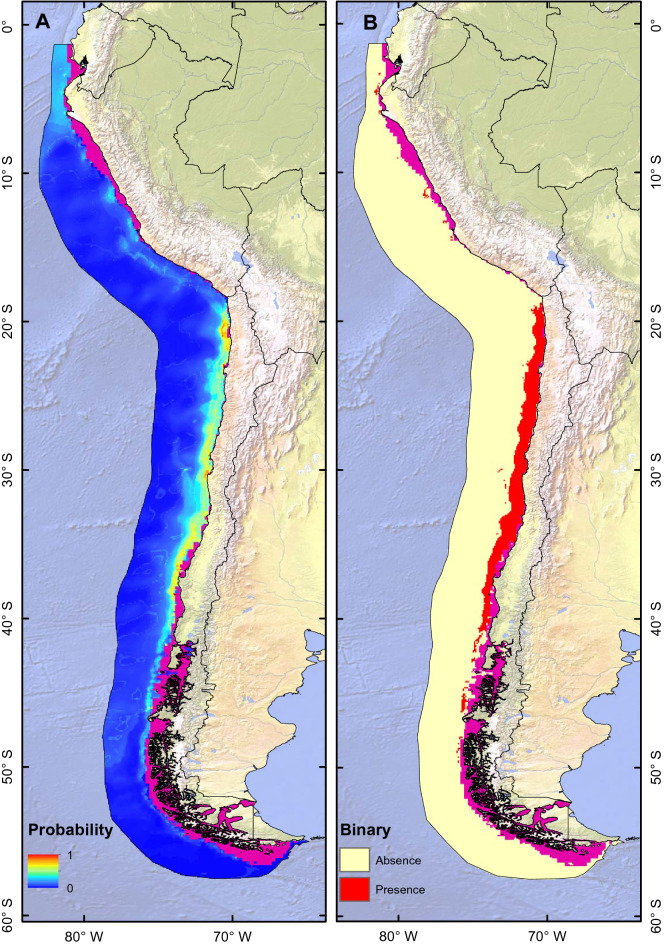
Potential current distribution maps generated through the Ensemble Small Models (ESM) approach for *Calanus chilensis* in the 200–400 m depth range of the Humboldt Current System. (**A**) Ensemble model suitability, (**B**) Ensemble binary model. Magenta color indicates depths greater than 200 m. This figure was generated using ArcGIS 10.4.1 (ESRI, Redland, CA; www.esri.com). Map projection is WGS84 (EPSG 4326).

The ESM for the 200–400 m depth range using the Copernicus database suggests that *C. chilensis* has a distribution restricted to the coast of Chile (Fig. 3A), which extends to the south beyond the range of its known distribution (Fig. 3A,B). The binary prediction revealed a continuous distribution from northern Chile (~ 18°S) to the Los Lagos region (~ 41° S), whereas further south, the probability of presence is discontinuous until 49° S (Fig. 3B). The performance of the ESMs showed high values of AUC and Continuous Boyce Index (CBI) (0.897 and 0.66 respectively), indicating the good performance of the models (Table 1). The variables that contributed the most to the ESM were Eddy Kinetic Energy (EKE) (0.263%), chlorophyll-a (0.253%), and Net Primary Productivity (NPP) (0.25%) (Table 2). The ranking of each technique until the final assemblage and the contribution of each variable are presented in Tables 1 and 2 respectively.

**Table 1:** Models’ performance of ensemble small models modelling (ESM) method for each technique runs and final ensemble forecasting (200–400 m depth range).

Model	Kappa	AUC	TSS	MPA	CBI
GAM	0.171	0.815	0.654	0.179	0.770
GBM	0.113	0.880	0.684	0.065	0.800
GLM	0.102	0.890	0.797	0.530	0.364
RF	0.287	0.802	0.382	0.025	0.780
Ensemble forecasting	0.121	0.897	0.805	0.236	0.664

*GAM* generalized additive model, *GBM* generalized boosted models, *GLM* generalized linear models, *RF* random forest, *AUC* area under curve, *TSS* true skill statistic, *MPA* minimum predicted area, *CBI* continuous boyce index.

**Table 2:** Variable contribution (weighted means) for each modeling technique used in ESM and final ensemble projections (200–400 m depth range).

	GAM	GBM	GLM	RF	Ensemble
Chlorophyll *a*	0.282	0.253	0.242	0.238	0.253
Eddy kinetic energy	0.212	0.247	0.314	0.271	0.263
Net primary productivity	0.279	0.247	0.218	0.267	0.250
Salinity	0.227	0.253	0.226	0.225	0.233

*GAM* generalized additive model, *GBM* generalized boosted models, *GLM* generalized linear models, *RF* random forest.

The ESM model approach for the 200–400 m depth range using the bio-ORACLE database indicated a high performance of the model (AUC 0.955, TSS 0.914, and CBI 0.841) (Table S1). The geographic prediction indicated a lower predicted probability in relation to the upper bathymetric range (Fig. S2). The binary prediction of the ESM revealed a more heterogeneous and fragmented distribution in deeper layers (Fig. S2). The variable contribution for each modeling technique used in ESM and final ensemble projections indicated that Eddy Kinetic Energy (EKE) and Net Primary Productivity (NPP) (Table S2) are the main drivers of *C. chilensis,* generally coinciding with the prediction generated based on the variables of the Copernicus database (Table 2).

### Projections to future scenarios

As shown in Table 3, the models indicate that under a low-impact scenario (Representative Concentration Pathway 2.6 (RCP2.6) there would be a smaller number of lost areas in relation to a high-impact scenario (RCP8.5). Similarly, under benign scenarios there is a greater number of gained areas; that is, pixels which are currently not occupied by the given species but predicted to be in the future; however, in the long term (years 2090–2100), the pixel gain is less. Regarding the percentage of gain (percentage of new sites considering the species’ current distribution size) and loss (percentage of currently occupied sites to be lost), there is a greater gain of areas only in low-impact scenarios and in the short term, whereas in the long term the gain decreases and the loss of areas increases. The general result of the projection (species range change) revealed a generalized loss of areas in the geographic distribution range both in low and high-impact scenarios, mainly for the long term (Fig. S3, Table 3).

**Table 3:** Predicted loss, expansion, and areas of no change (in pixels, resolution =  ~ 9.2 km) for the distribution of *C. chilensis* into the near future (2040–2050) and far future (2090–2100) and under scenarios of low (RCP2.6) and high impact (RCP8.5).

	Near future (2040–2050)		Far future (2090–2100)	
	RCP2.6	RCP8.5	RCP2.6	RCP8.5
Loss	431	690	921	1503
Stable 0	24,294	24,450	25,008	25,379
Stable 1	9522	9263	9032	8450
Gain	1417	1261	703	332
Percentage loss	4.3	6.9	9.2	15.1
Percentage gain	14.2	12.6	7.1	3.3
Species range change	9.9	5.7	− 2.19	− 11.7
Current range size	9953	9953	9953	9953

Loss = corresponds to the percentage of currently occupied sites to be lost. Stable 0 = number of pixels which are not currently occupied by the given species and not predicted to be. Stable 1 = number of pixels currently occupied by the given species, and predicted to remain occupied into the future.

MESS analysis identified areas where no analogs or novel climates were present. Dissimilarity values were relatively low within suitable areas for *C. chilensis*, not showing severe extrapolation from the models (Fig. S4).

## Discussion

Given that the distribution of *C. chilensis* is explained by a reduced number of oceanographic variables, linked to productivity (chlorophyll-a) and physical variables such as MLD, our model (Fig. 1) is consistent with that reported in the literature. Indeed, large calanoid species that are common in shelf waters were more abundant within nearshore eddies rich in chlorophyll-a and other cyclonic eddies far offshore^55^. These environmental features are present in water mass typical of intensive upwelling^13^. According to Morales et al.^55^ the eddy field alone did not explain the geographic distribution of *C. chilensis* at mesoscale^55^, where the availability of chlorophyll-a and waters with low salinity is key to explaining the distribution of this species.

Historically, *C. chilensis* is reported in the HCS all along the Chilean and Peruvian coast from approximately 21 to 45°S, with no data available for more southern or northern regions^13^. In earlier studies conducted in Chilean waters, *C. chilensis* was found inhabiting oxygenated waters over the oxygen minimum zone (OMZ)^56^, although sometimes smaller portions of the population were also found below the oxycline^57^, through passive sinking^58^, diel vertical migration (DVM)^59,60^, or combining DVM (daily vertical migration) and predation. The latter and the fact that this species is restricted to oxygenated waters above the OMZ^56^, represents an important food resource for the larger animals populating this zone, makes *C. chilensis* a species of ecological importance in this system. It is also characterized by being the only species of the family Calanidae where not only a resting stage can tolerate hypoxia^13^, and, due to its large size and abundance, *C. chilensis* play an important role for the carbon flux into the OMZ^13^, due to very active diel vertical migration^57^.

Geographic predictions by the Bayesian BART method for the range 0–200 m indicate that *C. chilensis* is distributed latitudinally from 1 to 55°S in areas near the coast (Fig. 1). Similarly, several studies off Chile found *C. chilensis* close to the shore^61^ with the highest abundances observed (ca. 44.000 ind. m^2^) in a narrow band within Cold Coastal Water, which coincides with the Poleward Undercurrent^13^. In particular, a predicted high-probability zone lies further offshore in central Chile, where a high abundance of *C. chilensis* has been reported, which can be interpreted as the result of advection by eddies^55,61^. The probability of presence of *C. chilensis* in the HCS appeared to decrease with winds flowing from east to west (between 5° and 25°S) and increase with winds flowing from the west and its meridional components (between 30° and 60°S) (Fig. S5).

The model prediction for the bathymetric range (200–400 m; Fig. 3) revealed a pattern similar to that reported for the 0–200 range; however, there would be a greater geographic discontinuity. As *C. chilensis* is known for inhabiting the upper 250 m^13^, the occurrences reported at greater depths may be thus circumstantial or correspond to sink populations from higher strata that act within the context of a source-sink dynamic^62,63^. In this stratum, the variables that explain the spatial distribution were related to EKE and NPP. EKE reveals the presence of mesoscale eddies^64^; therefore, the distribution of *C. chilensis* is conditioned by kinetic energy, whereas chlorophyll-a as a proxy of productivity is also another important modulator of the presence of *C. chilensis* in deeper areas. The latter confirms that chlorophyll-a is the main factor affecting the distribution of most species in the HCS^55^, as, for example, large copepod species which are dominant in the upwelling area are associated with the presence of chlorophyll-a and cyclonic eddies^65^.

For the prediction of 0–200 m, the high probability in areas close to 35°S, may be due to a greater historical sampling effort and the wide distribution of copepod species over the shelf/slope in that region, which is explained by physical and biological mechanisms that could be acting to extend the productive area of the coastal upwelling zone^55^.

Although both the BART and the ESM models generally predict an almost continuous distribution from Ecuador to Magellan, the biological characteristics of *C. chilensis* indicate that this species, although it reproduces throughout the year, does so less frequently in central Chile^66,67^ due to the existing variability within the HCS, such as seasonality in food availability and advection, which are some of the main drivers of their seasonal occurrence and abundance^13^. This reveals that seasonal and mesoscale processes are important when interpreting model outputs on the geographic distribution of marine organisms, since such models do not incorporate these variables, as they are not available in repositories such as bio-Oracle or MARSPEC (e.g. upwelling regimes^68^).

The use of a Bayesian approach to model the distribution of *C. chilensis* represented a methodological advantage, because it allows quantifying the uncertainty of the prediction^69^. In general, the use of Bayesian spatial models can help in the analysis of data with geographically uneven levels of sampling effort^70^, a common situation in biodiversity data^71^, and especially in ecosystems such as the HCS. Therefore, given the presence of bias in the data, the most suitable analysis strategy is the use of approximations that allow this bias to be reduced, reducing its influence on the parameter estimates^70^. Another difficulty when modeling the distribution of endemic species such as *C. chilensis* is obtaining enough occurrences^72–74^, which is a recurrent situation for species that are rare, endemic or with biased sampling^75^. To take into account the low number of occurrences for the 200–400 m strata, we used ESMs, an approach described to date as the most suitable for getting robust predictions even when modeling rare species or with a reduced number of occurrences^76,77^.

In a future scenario (medium and long term), *C. chilensis* would experience a drastic reduction in its geographic range, because of its high dependence on oceanographic conditions which may change with the global warming going on, mainly the availability of nutrients, variations in salinity, and depth of the mixed layer (see Fig. 2). In this regard, this species, being endemic to an ecoregion, with particular oceanographic characteristics, is more sensitive and would be mostly exposed to these fluctuations in the future. However, this trend would be greater in the models that simulate the “worst” climate scenario, and in the long term (2090–2100), being less affected by environmental variability in the short term and under “benign” scenarios (e.g. RCP26; see Table 3). These reported trends are consistent with the effects that anthropogenic global climate change would produce, mainly with respect to changes or modifications in the latitudinal distribution ranges^23,78^. Whatever, the case, altered distribution of this species, may have important ecological and biogeochemical consequences for the functioning of the HCS. *C. chilensis* is a key secondary producer in the upwelling zone^11^, and has an important role in nutrient recycling and downward flux of C^57^. Constrained populations may thus affect the food web structure and have implications for the C cycling of the upwelling system.

In this study, we demonstrated that the distribution of *C. chilensis* is conditioned by productivity and mesoscale events which drive its spatial distribution in the HCS. In other regions of the world ocean, predicted changes in productivity, driven by global warming, are expected to alter the distribution of secondary producers over large spatial scales^79,80^, including dominant copepod species, such as *C. finmarchicus* in the North Atlantic^81^ and *Centropages typicus* in coastal areas of the Atlantic Ocean^82^. However, over regional scales, the maintenance mechanism used by *C. chilensis* and probably many other endemic zooplankton species^83^ present in coastal upwelling systems certainly needs further investigation.

Our model results and predictions are certainly subject to potential biases, derived from data limitations (sampling gaps), and other processes occurring over spatial–temporal scales smaller than model resolution. For instance, the mesoscale and sub-mesoscale advective processes may influence the distribution of drifting planktonic copepods^55^. Also, the spatial arrangement of metapopulations may be subject to differential responses to a highly heterogeneous environment, and so producing distinctive patches of the species over mesoscale to large-scale domains^2^.

## Methods

### Study area

The study area comprised the Humboldt-Current large marine ecosystem ranging from Peru to the southern zones of Chile^84^. These regions encompass the currently known geographic distribution of *C. chilensis*^6^. However, we extended the northern limit of the HCS by three degrees to consider new occurrences off the coast of Ecuador. Since the extension of the geographical area can influence the performance of SDM^85^, the spatial scale of the study area must consider the dispersal capacity of the target species^26^. To address this, we considered the HCS given that *C. chilensis* has been described as endemic to this ecosystem^66^ and it is also restricted to the upwelling zone^10,11,86^. These areas were considered to be accessible world (M) according to the theoretical framework proposal of Soberón & Peterson^87^.

### Occurrences and quality control procedures

We examined 270 records of *C. chilensis* obtained from the Ocean Biodiversity Information System (OBIS) and Global Biodiversity Information Facility (GBIF), as well as records available in specialized literature. All the above online databases were accessed on October 15, 2021 (Suppl. Table S3). Species occurrence records were obtained using the robis^88^ and rgbif packages^89^ implemented in the R software [Ref.^90^]. After retrieving the data, we eliminated records without information on the geographic coordinates, coordinates equal to zero, or records located inside continents. We selected only records at the level of species and excluded duplicate records. In addition, to avoid spatial biases in the sampling effort, which are common when literature and databases are used^75^. The temporal resolution of the species occurrences data was from the years 1960 to 2021. We carried out a spatial thinning approach to eliminate records with a minimum distance of 5 km from each other using the spThin R package^91^.

From the 82 thinned occurrence records of *C. chilensi*s, models were made for two bathymetric ranges: 0–200 m and 200-400 m. For 0–200 m depth range, 51 occurrences were obtained; and, after spatial thinning, a total of 24 records were recovered. For 200–400 m depth range, 31 occurrences were obtained; and, after spatial thinning, a total of 8 records were recovered (Fig. S6). The occurrences of *C. chilensis* are available in Supporting Information Table S3 and the Figshare repository https://doi.org/10.6084/m9.figshare.19747618.v1.

### Environmental database

The remotely sensed satellite data used for analyses were obtained from Copernicus Marine Environment Monitoring Service (https://marine.copernicus.eu/) to a resolution of 0.083 and 0.25 degrees. The temporal resolution of the data was 1993–2019. Each environmental layer represents annual average values per cell and corresponds to an integrated average from 0 to 200 m and 200–400 m depth respectively. Variables with a resolution of 0.25 degrees were resampled using Cubic Convolution Interpolation in ArcGIS 10.4.1 [Ref.^92^].

We downloaded seven variables that have a close relationship with the biology of *C*. *chilensis*^50^: chlorophyll-a (mg m^−3^), dissolved oxygen (mmol m^−3^), MLD (m), Net Primary Productivity (NPP) (mg m^-3^ day^−1^), pH, salinity and temperature (°C). The Eddy Kinetic Energy (EKE) corresponds to the Sea Level Anomaly intensity and was calculated by its impact on the upwelling^7,12^. Surface data in terms of geostrophic sea water velocity for the zonal (east-speed U) and meridional (north-speed V) components were extracted from the Copernicus database. Calculations were made using the formula, EKE = 1/2x(U2 + V2), expressed in cm^2^/s^2^.

Considering that the correlation between variables may affect the performance of SDM^93,94^, we used the variance inflation factor (VIF) to detect collinearity between predictors. Using the usdm package^95^, variables with a VIF > 3 were excluded^96^. Complementarily, we evaluated the correlation and eliminated variables with Spearman’s rank correlation coefficient (ρ > 0.7). Thus, the variables used for posterior analyses were chlorophyll-a, MLD, EKE and salinity (See Supplementary Figs. S7, S8 and Table S4).

### Species distribution modelling

To evaluate the potential distribution of *C. chilensis* based on oceanographic predictors, we used two approaches, BART and ESM for 0–200 m and 200–400 m depth ranges respectively, given the larger number of occurrences available for the former and the fewest occurrences available for the latter.

BART is a tree-based method of machine learning which is based on a Bayesian approach to classification and regression trees (CART). BART is defined by a prior distribution and a likelihood for returning occurrence predictions that enable the quantification of uncertainty around the predictions and the estimation of the marginal effects of the covariates^69^. The BART method is a technique that deals with non-linear and non-monotonic relationships between response and predictor variables, and allows estimating the probability of presence of a species or its populations. BART models were run with default parameters using 200 trees and 1000 back-fitting Markov chain Monte Carlo (MCMC) with 90 iterations, discarding 20% as burn-in through embarcadero R package^69^. Model performance was evaluated using the AUC of the receiver operating characteristic curve (ROC) and True Skill Statistics (TSS). To estimate the potential distribution of *C. chilensis*, the predictions (i.e. probability of presence) were converted into binary predictions using a threshold at which TSS is maximized (max TSS)^97,98^. The Bayesian spatial method allows the incorporation of spatial correlation of the variables and the uncertainty of the parameters in the modeling process, resulting in a better quantification of the uncertainty (credible intervals)^99,100^. Bayesian spatial models may also aid data analyses with geographically uneven levels of survey effort, which reduces its influence on estimates of the effects of environmental variables^70^.

Given that accurate species distribution models require a minimum occurrence record^73^, we used the ESMs’ approach to identify suitable areas for the presence of *C. chilensis* and estimate its geographic distribution at the 200–400 m depth range, since it is suitable for species with a low number of occurrence records^76,77^. The ESMs’ method is a technique that allows obtaining statistically robust habitat suitability models from combinations of bivariate models. It has been proved that this method can characterize reliable suitability models with less than 25 occurrence records^76^. The final ESM projection assembly was obtained by calibrating four modeling techniques, generalized additive model (GAM), generalized linear model (GLM), generalized boosted regression modeling (GBM) and random forest (RF). We selected default model tunings within ecospat package^101^. For each ESMs’ run, the presences were partitioned into 70% for training and 30% for testing. We use 1000 random pseudo-absences in the calibration and projection areas; the prediction ensemble was obtained by averaging 10 runs weighted according to the AUC values. To calibrate and project the ESMs, four uncorrelated environmental predictors were used to model the distribution of *C. chilensis*: chlorophyll-a, MLD, EKE and salinity (Supplementary Table S4, Fig. S8). Finally, the projected ESMs were transformed into binary prediction using the minimum predicted area (MPA)^102^.

Model performance of ESM was evaluated using the multi-metric approach to determine the variability among estimates, the AUC of the ROC, TSS, Cohen's kappa, and the Continuous Boyce Index (CBI). The AUC ranges from 0 to 1, where scores lower than 0.5 indicate discrimination worse than random, and a 1 score indicates perfect discrimination^103^. TSS ranges from − 1 to + 1, where + 1 indicates perfect agreement between predictions and observations, and values of 0 or less indicate agreement no better than random classification. Cohen's kappa statistic ranges from − 1 to + 1, where + 1 indicates perfect agreement and values of zero or less indicate a performance no better than random^97^. Finally, the CBI varies from − 1 for an inverse model to 0 for a random model to 1 for a perfect model^104,105^. The analyses were performed in the ecospat package^101^.

In addition to the models fitted using the oceanographic layers retrieved from the Copernicus database, we fitted a second set of SDMs using layers downloaded (except for MLD and EKE) from the bio-ORACLE database version 2.2^77^ at a resolution of 0.08 degrees (~ 9.2 km at the equator). Then, to evaluate the variability in model predictions depending on the used environmental dataset, outputs resulting from the BART and ESM models based on Copernicus and bio-ORACLE data were compared through Schoener's D overlap index, which ranges from 0 (no overlap) to 1 (complete overlap). Analyses were carried out with ENMTools package^106^ in R [Ref.^90^]. Results indicated a high similarity between predictions for the 0–200 m depth range (BART approach) (D = 0.86; Spearman rank correlation = 0.81), and a lower similarity between predictions for the 200–400 m depth range (ESM approach) (D = 0.69; Spearman rank correlation = 0.42). For this, we present the models built with the variables from the Copernicus database, as they can be downloaded separately for distinct depth ranges, which is not possible with the bio-ORACLE database. The BART and ESM models for the depth range of 0–200 and 200–400 m built with bio-ORACLE variables are shown in Supplementary Fig. S1, S2, Table S4.

### Projection of models to the future

To identify future potential shifts in the distribution range of *C. chilensis* we modeled the distribution in the context of global climate change, projecting the models in two periods: the near future (2040–2050) and the far future (2090–2100). In both scenarios, two representative concentration pathways (RCP) scenarios were used, RCP2.6 and RCP8.5. RCP2.6 represents a peak-and-decline scenario ending with very low greenhouse gas concentration levels by the end of the twenty-first century, whereas RCP85 is a scenario of increasing emissions over time leading to high greenhouse concentration levels (see Ref.^107^). To assess the extrapolation risk, we performed a Multivariate Environmental Similarity Surface (MESS) analysis to determine novel climatic conditions under future climate scenarios^108^. Negative values indicate localities that are environmentally dissimilar from the reference region. Positive values indicate climate similarity^108^. The MESS analyses were performed in ntbox package^109^. The projections were made using the BART approach only for the 0–200 m, given the greater availability of occurrence records. Since variables such as MLD and EKE are not available for the future, we model using salinity, chlorophyll-a and keeping MLD constant, since they correspond to the variables selected as the most important to explain the geographic distribution in the present (Fig. 2). The oceanographic variables were obtained from bio-ORACLE v 2.1^107^ at a resolution of 5 arcminutes (~ 9.2 km) through the SDMpredictors package^110^. We used a TSS threshold to convert maps into binary presence/absence to calculate changes in the size of suitable habitats using biomod2 package^111^.

We followed best-practice standards in SDM regarding guidelines for response and predictor variables, model building and evaluation^112^. We provide as Supplementary Table S5 a description of the modeling steps following the ODMAP (Overview, Data, Model, Assessment and Prediction) protocol^112^. The geographic data were processed in ArcGIS 10.4.1 [Ref.^92^] and all statistical analyses were performed in the R 4.0.1 [Ref.^90^].

## Supplementary Information

Supplementary Information.

## Data Availability

The datasets generated analysed during the current study are available in the Figshare repository https://doi.org/10.6084/m9.figshare.19747618.v1.
