# Supplementary material for: Modeling present and future distribution of plankton populations in a coastal upwelling zone: the copepod Calanus chilensis as a study case
Source: Sci Rep. 2023 Feb 23;13:3158. doi: 10.1038/s41598-023-29541-9 (PMC9950369; doi:10.1038/s41598-023-29541-9)
Supplement: Supplementary file 1 — Supplementary Information. [file 41598_2023_29541_MOESM1_ESM.docx]

Supplementary Information

**Modeling present and future distribution of plankton populations in a coastal upwelling zone: the copepod Calanus chilensis as a study case.**

Reinaldo Rivera^1*^, Rubén Escribano^1,2,^ Carolina E. González^1^ and Manuela Pérez-Aragón^1,2^

^1.^ Millennium Institute of Oceanography (IMO), University of Concepcion, Concepcion, 4030000, Chile.

^2.^ Department of Oceanography, Faculty of Natural and Oceanographic Sciences, University of Concepcion, Concepcion, 4030000, Chile.

* Corresponding author e-mail: reijavier@gmail.com

**Supplementary Table S1.** Values of model performance metrics for each ESM (Ensembles of Small Models) with variables from the bio-ORACLE database. (200 – 400 m depth range). GAM= Generalized Additive Models, GBM= Gradient Boosting Machine, GLM= Generalized Linear Model, RF= Random Forest, AUC= area under the ROC curve, TSS= true skill statistic, MPA= minimum predicted area, CBI= Continuous Boyce Index

| Model | Kappa | AUC | TSS | MPA | CBI |
| --- | --- | --- | --- | --- | --- |
| GAM | 0.277 | 0.876 | 0.721 | 0.157 | 0.393 |
| GBM | 0.208 | 0.929 | 0.881 | 0.125 | 0.773 |
| GLM | 0.157 | 0.924 | 0.876 | 0.601 | 0.759 |
| RF | 0.204 | 0.951 | 0.842 | 0.067 | 0.699 |
| Ensemble forecasting | 0.290 | 0.955 | 0.914 | 0.251 | 0.841 |

**Supplementary Table S2.** Variable contribution for each modelling technique (GAM, GBM, GLM, RF) used in ESM and final ensemble projections with variables from the Bio-ORACLE database (200 – 400 m depth range). EKE= Eddy Kinetic Energy, NPP= Net Primary Productivity.

|  | GAM | GBM | GLM | RF | Ensemble forecasting |
| --- | --- | --- | --- | --- | --- |
| Chlorophyll-a | 0.209 | 0.243 | 0.243 | 0.223 | 0.230 |
| EKE | 0.242 | 0.249 | 0.267 | 0.318 | 0.270 |
| NPP | 0.276 | 0.262 | 0.250 | 0.236 | 0.255 |
| Salinity | 0.273 | 0.246 | 0.240 | 0.224 | 0.245 |

**Supplementary Table S3.** Occurrences: This table is available in the Figshare repository DOI <https://doi.org/10.6084/m9.figshare.19747618.v1> or can be provided as csv file by request at [reijavier@gmail.com](mailto:reijavier@gmail.com)

| **Dataset name** | **Citation** | **License** | **Records** | **Source** |
| --- | --- | --- | --- | --- |
| [Zooplankton of the Eastern South Pacific](https://obis.org/dataset/4b25fd6e-e5de-4866-aee5-2d7ea4c407da) | Escribano, R., Hidalgo, P., Manríquez, K., 2005. Humboldt Current species. | Unrestricted | 111 | OBIS |
| [Copepodos Chile](https://obis.org/dataset/75da81ec-cdd9-4bbc-aaeb-c499df50fe68) |  | This work is licensed under a Creative Commons Attribution Non Commercial (CC-BY-NC) 4.0 License | 68 | OBIS |
| [copepods_rvsonne_2010 Chile](https://obis.org/dataset/e18538d7-87e2-470d-8848-7a6b76394bde) | Fierro P A (2018): copepods_rvsonne_2010 Chile. v1.5. Universidad de Concepción e Instituto Milenio de Oceanografía (IMO). Dataset/Samplingevent. http://ipt.iobis.org/esp-obis/resource?r=copepods_rvsonne_ocurrence&v=1.5 | This work is licensed under a Creative Commons Attribution Non Commercial (CC-BY-NC) 4.0 License | 4 | OBIS |
| [NIWA Invertebrate Collection](https://obis.org/dataset/2c6db58f-ae91-4a17-9f0f-7db28506b94f) | NIWA (2018): NIWA Invertebrate Collection. v1.1. The National Institute of Water and Atmospheric Research (NIWA). Dataset/Occurrence. https://nzobisipt.niwa.co.nz/resource?r=obisspecify&v=1.1 | This work is licensed under a Creative Commons Attribution (CC-BY) 4.0 License | 2 | OBIS |
| [ZooGene A DNA Sequence Database for Calanoid Copepods and Euphausiids](https://obis.org/dataset/36b58f28-0a03-4447-b688-e4eed56afa3d) | Zooplankton genomic database (ZooGene) project: integrating molecular, taxonomic, and oceanographic data. Bucklin,A.; Wiebe,P. H.; Frost,B. W.; Groman,R. G.; Fogarty,M. J. | Unrestricted | 2 | OBIS |
| [Copepoda of the Equatorial Eastern Pacific](https://obis.org/dataset/7694fdc5-fd4e-4106-ad20-edc046cc8618) | Tutasi, P. 2005 Copepods Equatorial Eastern Pacific | This work is licensed under a Creative Commons Attribution Non-Commercial (CC-BY-NC) 4.0 License | 2 | OBIS |
| [National Museum of Natural History Invertebrate Zoology Collections](https://obis.org/dataset/89e23fc8-3f61-4480-9de3-358fe6eefe0b) | National Museum of Natural History, Smithsonian Institution NMNH Invertebrate Zoology Collection Database. National Museum of Natural History, Smithsonian Institution, 10th and Constitution Ave. N.W., Washington, DC 20560-0193, 2001, Version 3.2.04 (0802221). | This work is licensed under a Creative Commons Attribution (CC-BY) 4.0 License | 2 | OBIS |
| INSDC Sequences |  |  | 3 | GBIF |
| Copepoda of the Equatorial Eastern Pacific | Tutasi, P. 2005 Copepods Equatorial Eastern Pacific |  | 2 | GBIF |
| NIWA Invertebrate Collection | NIWA (2018): NIWA Invertebrate Collection. v1.1. The National Institute of Water and Atmospheric Research (NIWA). Dataset/Occurrence. https://nzobisipt.niwa.co.nz/resource?r=obisspecify&amp;v=1.1 |  | 3 | GBIF |
| NMNH Extant Specimen Records | Orrell T, (2021): NMNH Extant Specimen Records. v1.46. National Museum of Natural History, Smithsonian Institution. Dataset/Occurrence. https://collections.nmnh.si.edu/ipt/resource?r=nmnh_extant_dwc-a&v=1.46 |  | 3 | GBIF |
| Copepodos Chile |  |  | 68 | GBIF |
| International Barcode of Life project (iBOL) | Institute of Biodiversity, Animal Health and Comparative Medicine, College of Medical, Veterinary and Life Sciences, University of Glasgow (2016). International Barcode of Life project (iBOL). Occurrence dataset https://doi.org/10.15468/inygc6 accessed via GBIF.org on 2019-06-13. |  | 1 | GBIF |

**Supplementary Table S4**. VIF analysis for selection of environmental variables. EKE= Eddy Kinetic Energy, MLD= Mixed Layer Depth, NPP= Net Primary Productivity.

|  |  |  |
| --- | --- | --- |
| Depth | 0 - 200 |  |
| Database | Copernicus |  |
|  | **Variables** | **VIF** |
|  | Chlorophyll-a | 1.247 |
|  | EKE | 2.001 |
|  | MLD | 1.546 |
|  | Salinity | 2.024 |
|  |  |  |
| Depth | 0 - 200 |  |
| Database | Bio-ORACLE |  |
|  | **Variables** | **VIF** |
|  | EKE | 1.776 |
|  | MLD | 1.709 |
|  | NPP | 1.473 |
|  | Salinity | 1.700 |
|  |  |  |
| Depth | 200 - 400 |  |
| Database | Copernicus |  |
|  | **Variables** | **VIF** |
|  | Chlorophyll-a | 1.541 |
|  | EKE | 1.254 |
|  | NPP | 1.333 |
|  | Salinity | 1.236 |
|  |  |  |
|  |  |  |
| Depth | 200 - 400 |  |
| Database | Bio-ORACLE |  |
|  | **Variables** | **VIF** |
|  | Chlorophyll-a | 7.410 |
|  | EKE | 1.712 |
|  | NPP | 7.070 |
|  | Salinity | 2.556 |

**Supplementary Table S5.** ODMAP (Overview, Data, Model, Assessment and Prediction) protocol

| **ODMAP element** | **Contents** |
| --- | --- |
| **OVERVIEW** | |
| Autorship | Authors: Reinaldo Rivera Jara  Contact email: [reijavier@gmail.com](mailto:reijavier@gmail.com)  Title: Current and Future Influence of environmental drivers over the endemic species of the Humboldt system *Calanus chilensis* (Brodsky, 1959) (Calanoida: Calanidae). |
| Model objetive | **Objetive:** Mapping/interpolation  **Target output:** Maps of relative probability of presence |
| Taxon | **Single species:** *Calanus chilensis* (Brodsky, 1959) (Calanoida: Calanidae). |
| Location | Humboldts’s system |
| Scale of analysis | **Spatial extent (Lon/lat):** -83.021, -64.854, -57.512, -1.345 (xmin, xmax, ymin, ymax), covering 2.622.980 Km^2^. (South America Albers Equal Area Conic)  **Spatial resolution:** 0.08 degree  **Temporal extent/time period:** 1993 -2019  **Type of extent boundary:** irregular polygon |
| Biodiversity data overview | **Observation type:** database (OBIS and GBIF)  **Response/Data type:** Presence and background data. |
| Type of predictors | Oceanographic variables |
| Conceptual model /hypotheses | **Hypotheses about species-environment relationships:**  There is empirical evidence (Escribano & Hidalgo 2000; Escribano et al. 2008) that the oceanic variability of the upwelling zones influences the geographic distribution of *Calanus chilensis.* |
| Assumptions | **We assumed that:**  - Important ecological drives of the species distribution are included in the models (e.g., temperature, energy, productivity)  - The species is in (pseudo) equilibrium with its environment.  - The sample size is representative and the biases in the occurrences were considered and corrected. |
| SDM algorithms | **Algorithms:** We fitted Bayesian additive regression trees (BART)  and Ensembles of Small Models (ESM).  BART was chosen due to estimates the probability of a binary outcome based on a set of decision trees. BARTs have yet to be applied to SDM (Carlson 2020).  ESM was chosen due to competitive performance on small sample size.  **Model complexity:** Ensembles |
| Model workflow | 1. Search and cleaning of occurrence data. 2. Download and format of environmental data in GIS environment 3. Delimitation of study area (M sensu Soberón & Peterson 2005) 4. Training and calibration of models (BART and ESM) 5. Performance evaluation. |
| Software | **Software:** Analyses were conducted in R version 4.1.0 (R Core Team, 2020) and ArcGIS 10.4.1 (ESRI, 2016).  **Data availability:** occurrence data is available at figshare 10.6084/m9.figshare.19747618 |
| **DATA** | |
| Biodiversity data |  |
|  | **Taxon names:** single species: *Calanus chilensis* Brodsky, 1959  (AphiaID 346212 urn:lsid:marinespecies.org:taxname:346212)  **Ecological level:** population  **Data source:** OBIS and GBIF  **Sampling design:**  not applicable  **Sample size:** The raw data contains a total of 81 occurrences.  **Regional mask:** We clipped all data to the boundary of the study región (Humboldt Current System).  **Scaling:** Occurrence were spatially thinned (within 5 kilometers) using spThin R package (Aiello-Lammens et al. 2015).  **Data cleaning/filtering:** After cleaning and filtering the data, a reduced sample of 24 occurrences for 200-400 m depth and 8 occurrences for 200-400 m depth was obtained.  **Background data:** we generated 1000 random background.  **Error and biases:**  biases are accounted eliminating occurrences on land or outside the limits of the Humboldt Current System. |
| Data partitioning | 70% and 30% for calibration and evaluation (BART model) |
| Predictors variables | **Predictor variables:**  **Oceanographics:** chlorophyll a (mg m^-3^), dissolved oxygen (mmol m^-3^), Mixed Layer Depth (MLD) (m), net primary productivity (NPP) (mg m^-3^ day^-1^), pH, salinity (psu), temperature (°C) and Eddy Kinetic Energy (EKE)  **Data Sources:** Predictors were Copernicus ( <https://marine.copernicus.eu/>) and bio-ORACLE ( <https://www.bio-oracle.org>)  **Spatial resolution and extent of raw data:** Copernicus database (0.083 and 0.25 degree), bio-ORACLE Version: 2.2: 5 arcmin (ca. 0.088 or 9.2 km at the equator).  **Geographic projection:** proj= longlat, datum=WGS84  **Temporal resolution and extent of raw data:** Temporal resolution: 2000–2014.  **Extent of raw data:** -180, 180, -90, 90 (xmin, xmax, ymin, ymax)  **Data processing:** OD, NPP, Chl-a were downscaled to 0.08 degree using cubic interpolation |
| **MODEL** | |
| **Variable pre-selection** | |
| **Multicollinearity** | Multicollinearity was investigated using variance inflation factors (VIF) and Spearman rank correlation coefficients. |
| **Model Settings** | **Model settings:** From the set of candidate predictors, we estimated BART and ESM model.  **Model settings for extrapolation:** not applicable. |
| **Model estimates** | The "varimp" function returns variable importance in the BART model. En ESM model, the importance of each predictor was obtained as the average of each algorithm. |
| **Model averaging (Ensembles)** | The final ESM projection assembly was obtained by calibrating three modelling techniques (GLM, GBM, GAM and Random Forest) with the four variables. |
| **Non Independence** | The Bayesian spatial method allows the incorporation of spatial correlation of the variables and the uncertainty of the parameters in the modeling process, resulting in a better quantification of the uncertainty and accurate predictions (Ellison 2004). |
| **ASSESMENT** | |
| **Performance statistics** | Performance statistics estimated on training data. Model performance was assessed based on the true skill statistic (TSS), area under a receiver operating characteristic (ROC) curve (AUC) and Boyce index |
| **Plausibility checks** | **Response plot:** We used partial dependence plots to check the ecological plausibility of fitted relationships in BART models |
| **PREDICTION** | |
| **Prediction output** | Predictions of relative probability of presence expressed on a continuous and binary scale. |
| **Uncertainty quantification** | BART model explicitly showing the uncertainty associated with predicted species distributions.  We accounted for algorithmic uncertainty by applying an ensemble approach averaging over four different SDM algorithms for ESM. |


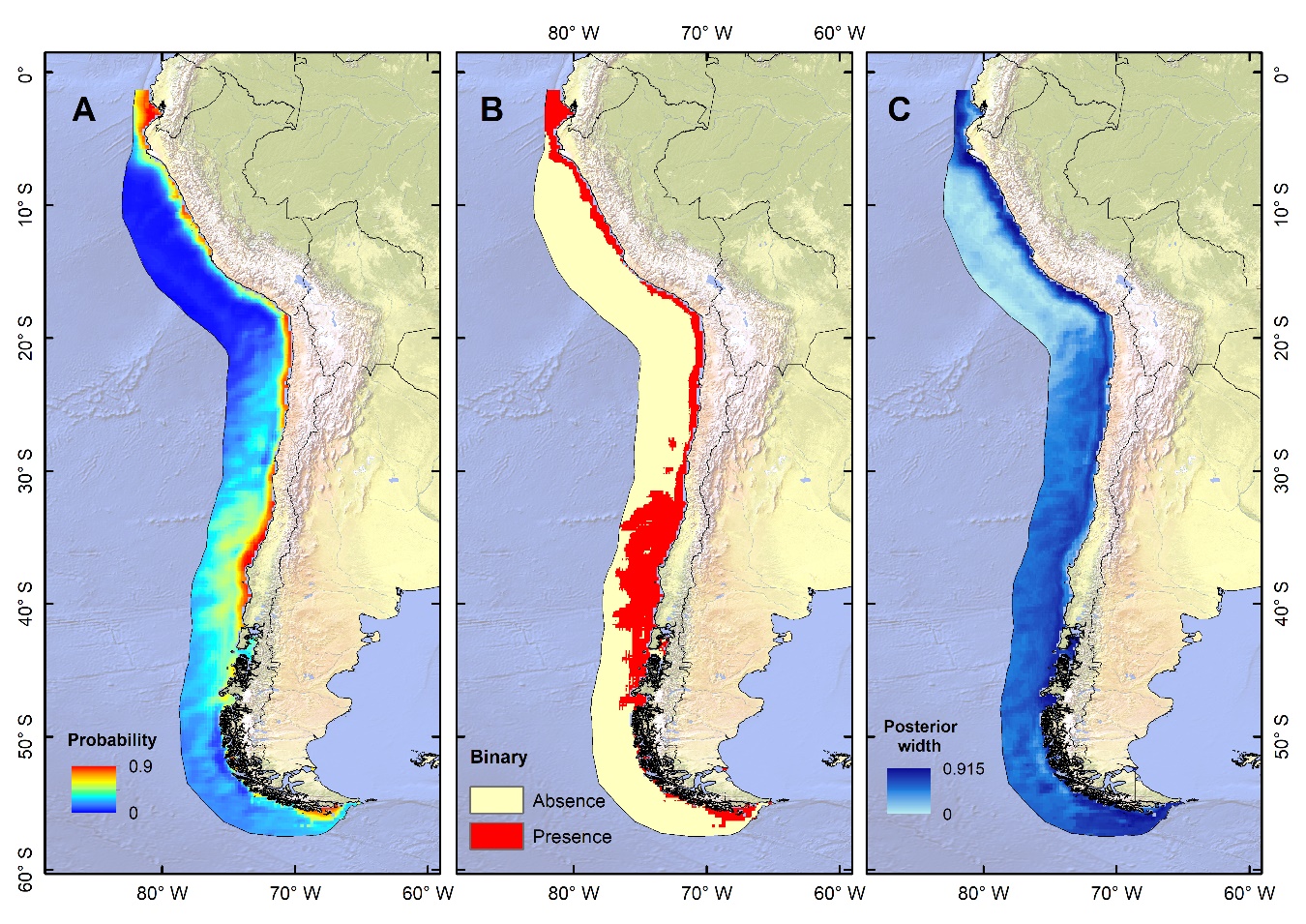


**Figure S1.** Median of the posterior probability of the presence (BART model) (A), binary model (B) and posterior width (95% credible interval) (C) for *Calanus chilensis* in the 0-200 m depth range of the Humboldt Current System using Bio-ORACLE database. Map projection is WGS84 (EPSG:4326).


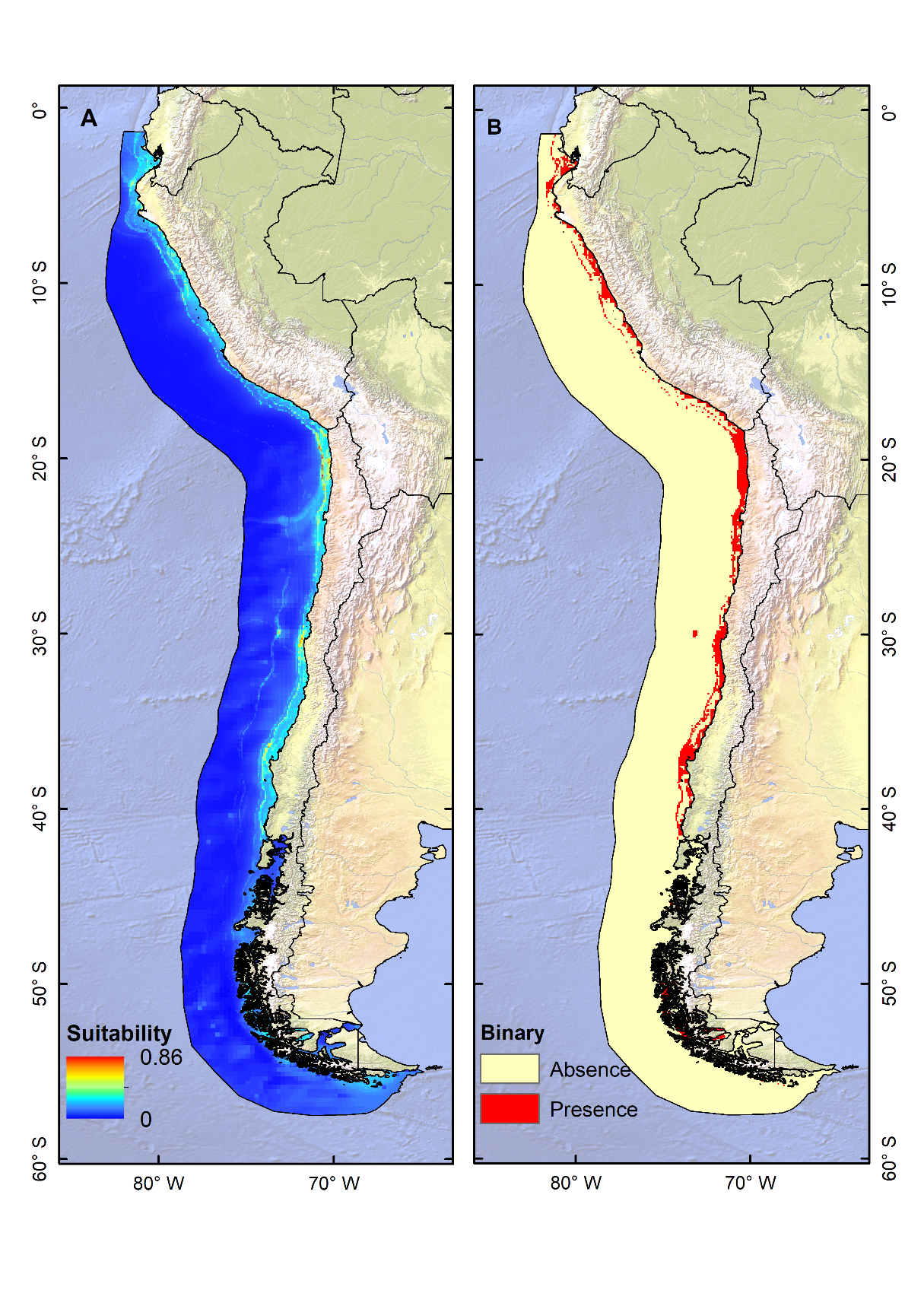


**Figure S2.** Ensemble model suitability (A) and ensemble binary model (B) for *Calanus chilensis* in the 200-400 m depth range of the Humboldt Current System using Bio-ORACLE database. Map projection WGS84 (EPSG:4326).


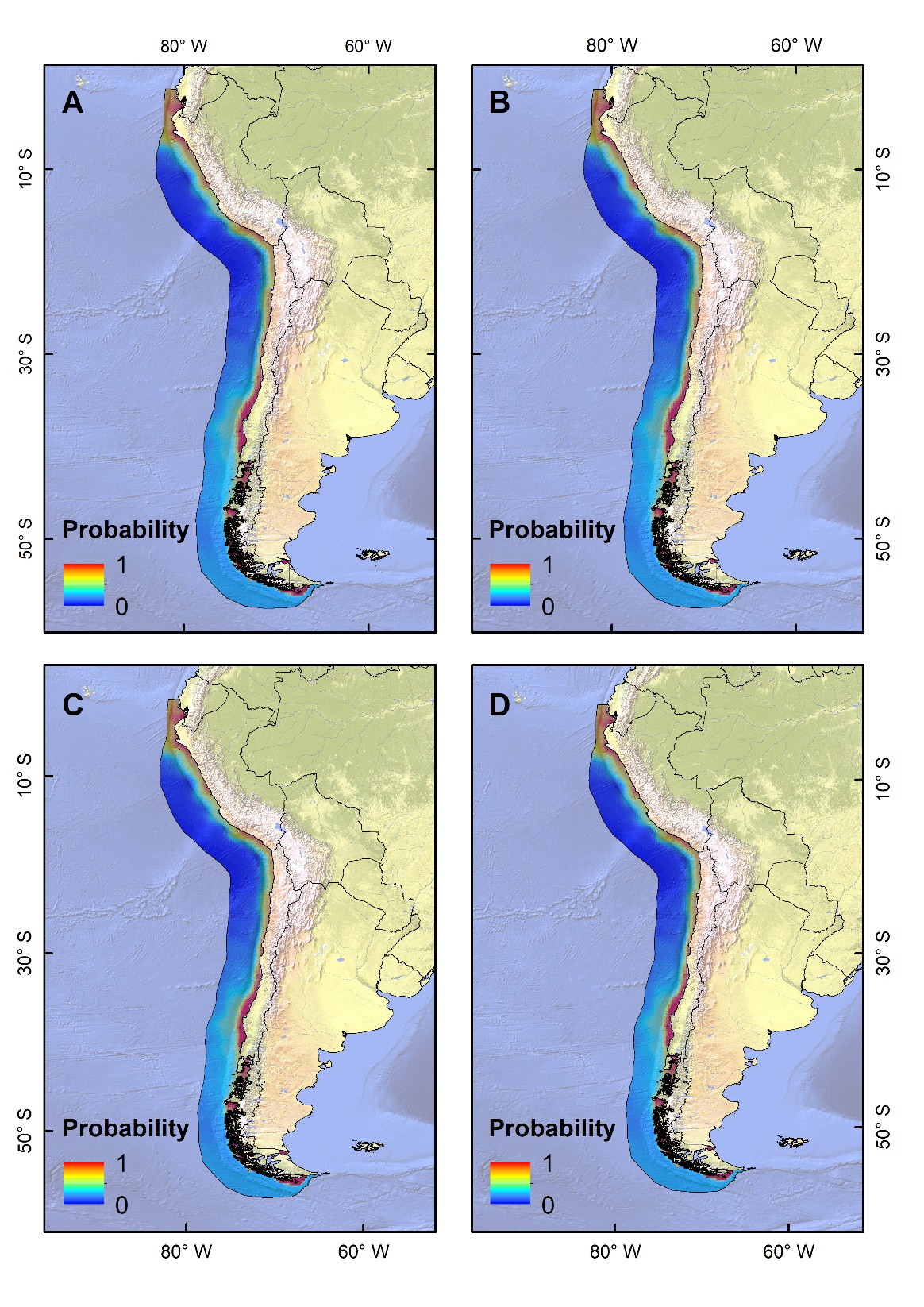


Figure S3. Habitat suitability maps for future climatic conditions predicted for 2040-2050 and 2090-2100. a) Map of habitat suitability for 2040-2050, RCP2.6 (‘best’ scenario), b) Map of habitat suitability for 2040-2050, RCP 8.5 (‘worst’ scenario), c) Map of habitat suitability for 2090-2100, RCP2.6, and d) Map of habitat suitability for 2090-2100, RCP 8.5. This figure was generated using ArcGIS 10.4.1 (ESRI, Redland, CA; www.esri.com). Map projection is WGS84 (EPSG 4326).


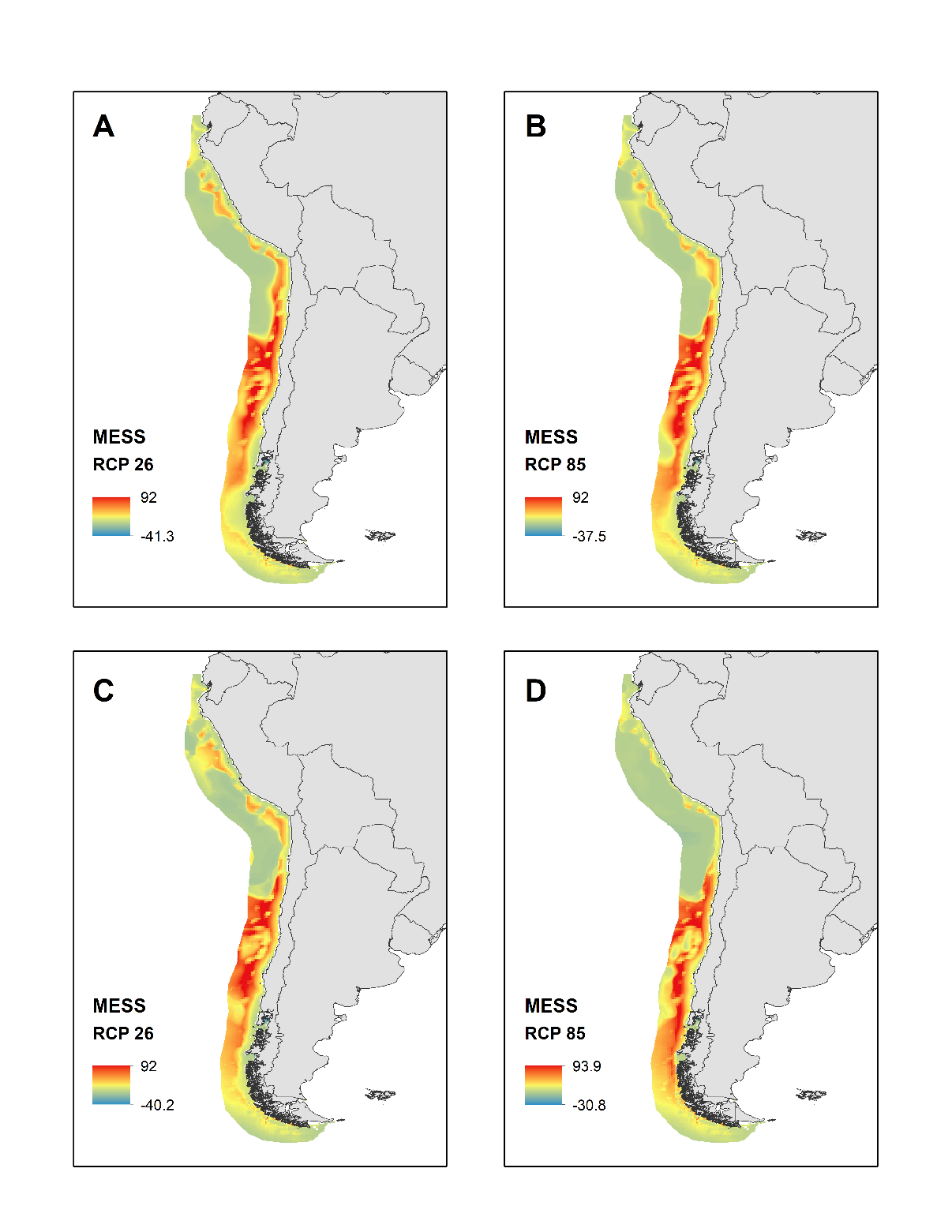


**Figure S4**. Results of the MESS indicating novel environments (i.e., climatic variables with different values from the recent climate). (A) MESS analysis for the years 2040-2050 (RCP 2.6), (B) MESS analysis for the years 2040-2050 (RCP 8.5), (C) MESS analysis for the years 2090-2100 (RCP 2.6), (D) MESS analysis for the years 2090-2100 (RCP 8.5).


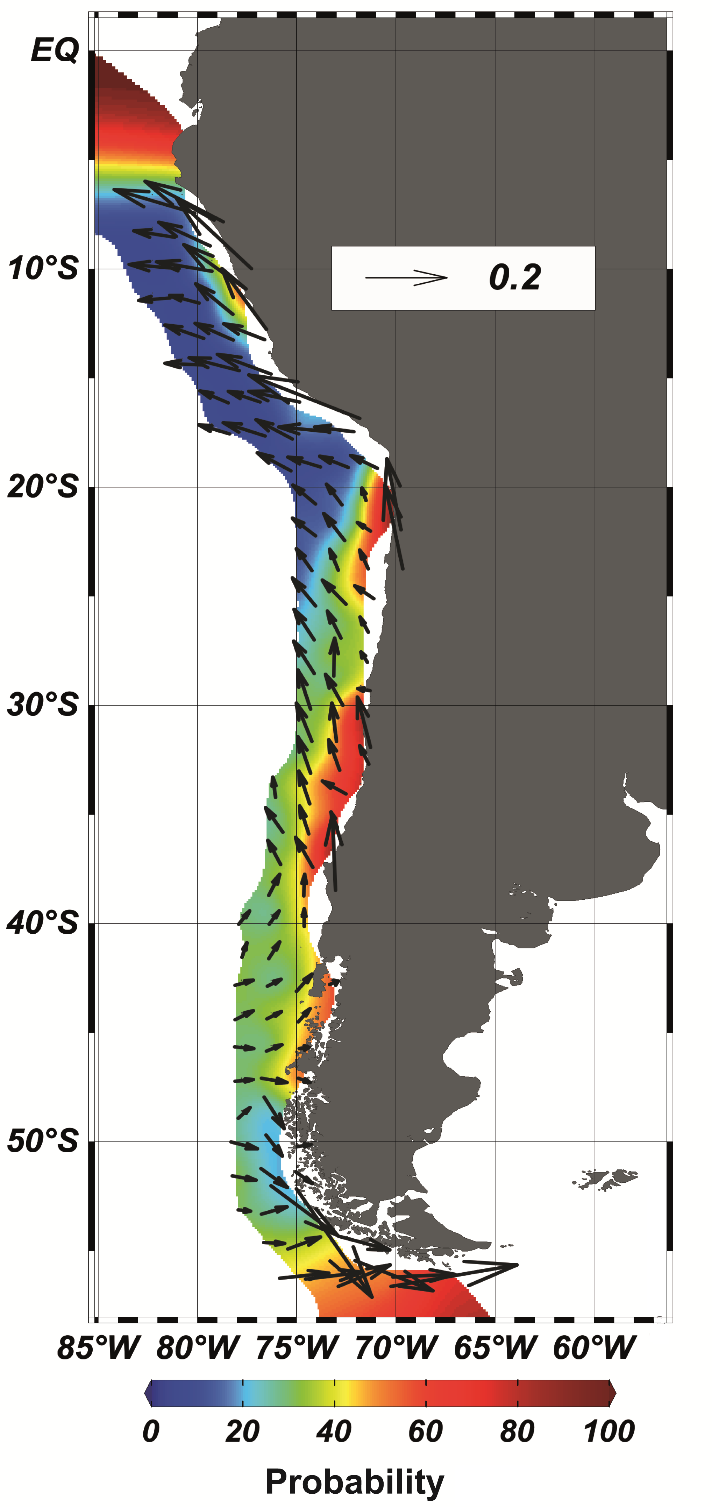


**Figure S5.** Probability of presence of *Calanus chilensis* with relation to average wind velocity in the HCS. This figure was generated using Ocean Data View (Schlitzer, R., Ocean Data View, <https://odv.awi.de>, 2018). Map projection is WGS84 (EPSG 4326).


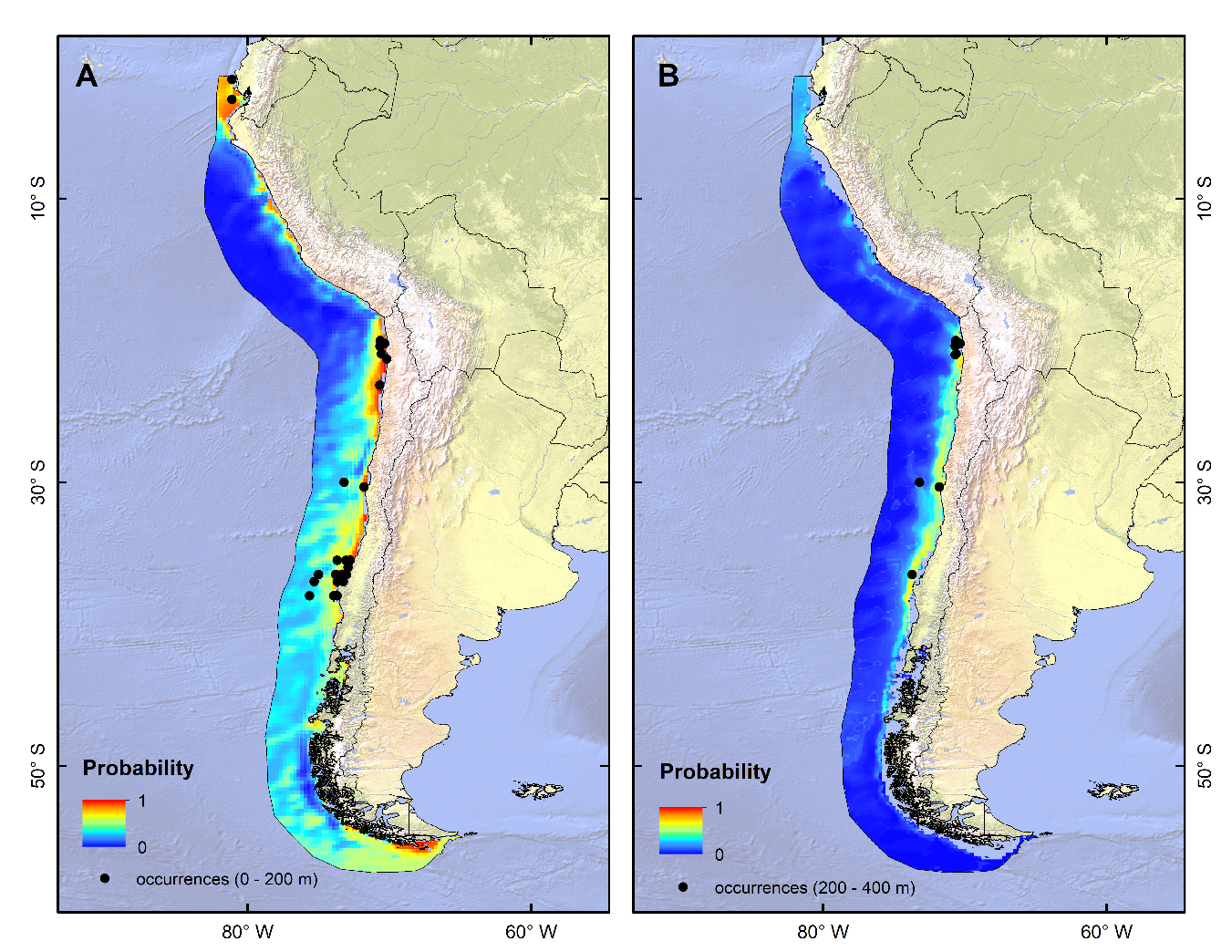


**Figure S6**. Occurrence records (thinned) and geographic prediction (expressed as probability) for *C. chilensis* using (A) BART model (0 -200 m), and B) ESM model (200 -400 m).


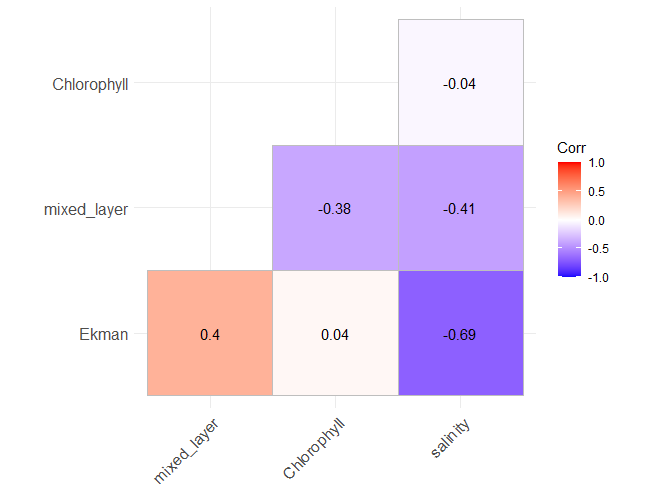


**Figure S7.** Spearman correlation matrix of environmental variables used in BART model, 0– 200 m depth range.


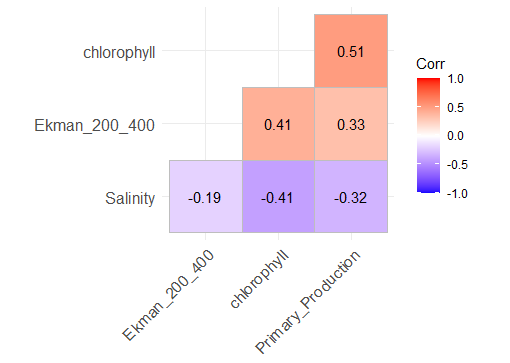


**Figure S8.** Spearman correlation matrix of environmental variables used in ESM models. 200-400 m depth range.
